# Effects of straw mulching practices on soil nematode communities under walnut plantation

**DOI:** 10.1038/s41598-020-72530-5

**Published:** 2020-09-18

**Authors:** Dagang Song, Akash Tariq, Kaiwen Pan, Wenkai Chen, Aiping Zhang, Xiaoming Sun, Yi Ran, Fanjiang Zeng

**Affiliations:** 1 Biogas Institute of Ministry of Agriculture and Rural Affairs, Chengdu, China; 2 Key Laboratory of Mountain Ecological Restoration and Bioresource Utilization & Ecological Restoration Biodiversity Conservation Key Laboratory of Sichuan Province, Chengdu Institute of Biology, Chinese Academy of Sciences, Chengdu, China; 3 Risk Assessment Lab of the Quality Safety of Biomass Fermentation Products, Ministry of Agriculture and Rurals Affairs, Chengdu, China; 4 University of Chinese Academy of Sciences, Beijing, China; 5 Xinjiang Key Laboratory of Desert Plant Roots Ecology and Vegetation Restoration, Xinjiang Institute of Ecology and Geography, Chinese Academy of Sciences, Urumqi, 830011 China; 6 State Key Laboratory of Desert and Oasis Ecology, Xinjiang Institute of Ecology and Geography, Chinese Academy of Sciences, Urumqi, 830011 China; 7 Cele National Station of Observation and Research for Desert-Grassland Ecosystems, Cele, 848300 China

**Keywords:** Biological techniques, Ecology, Microbiology, Plant sciences, Ecology, Environmental sciences

## Abstract

Agricultural management techniques such as mulching with crop straw can impact soil properties and may in turn change the structure and function of the soil food web. We investigated different straw mulching types and straw mulching coverage levels on soil nematodes community structure in walnut orchards. We set up a randomized experimental design with three straw mulch types, and three straw mulch distance treatments in a walnut plantation. The results indicated that the number of soil nematodes after straw mulching was lower than that found in the control (CK). However, the metabolic and structure footprints of the omnivore-predator nematodes showed higher values as compared to CK. The abundances of plant parasite and omnivore-predator nematodes were negatively correlated with ammonium nitrogen (NH^4+^–N) and dissolved organic nitrogen (DON), whereas soil moisture content (SM) had a negative correlation with the abundance of total nematodes. High structure index (SI), maturity index (MI) and low enrichment index (EI) values revealed a structured soil food web, medium soil enrichment, and fungal decomposition channel under the *mix straw* mulching treatments. Soil nematodes should be used as an indicator of soil functional changes resulting from straw mulching.

## Introduction

Crop straw has become an effective way to supplement soil nutrients and increase crop yield in modern agriculture because it is rich in various nutrients and physiologically active substances^1^. Straw mulching has important ecological significance for maintaining farmland fertility, reducing the use of chemical fertilizers, improving the carbon sink capacity of terrestrial soil, promoting the soil nitrogen cycle^2,3^, and reducing or avoiding environmental pollution caused by burning^4^.

In recent years, most studies of straw mulching have mainly focused on the physical, chemical and biological effects of soil and the physiological and ecological responses of mulched tree species to yield^5–7^. However, there is little research on straw mulching technology, and it has generally been performed on areas with extensive tree cover or in gardens; fine straw mulching technology has not been studied. In addition, straw mulching is mainly concentrated on food crops, and there are few studies of straw mulching in orchards. For walnut orchards, traditional management practices such as clean tillage cause serious soil erosion and reduced soil fertility, resulting in slow growth of walnut trees and reduced yield^8^. Therefore, it is necessary to consider using straw mulching to improve the sustainable development of walnut orchards.

With the growth of young walnut trees, the canopy width increases each year, so it is reasonable to designate the canopy radius at the coverage distance, considering the effect of canopy shading. In addition, we have previously reported that suitable straw mulching materials can promote the growth of walnut trees and increase the potential yield^9^. Therefore, it is an important step for the sustainable management of walnut orchards to determine suitable materials for straw mulch and location for straw mulch placement.

Soil nematodes are one of the most abundant metazoans on the earth. They exist widely in various habitats and play an important role in maintaining the stability of soil ecosystems, promoting material circulation and energy flow^10^. Nematodes are simple to extract and identify, and feed on diverse nutrient resources, making them very sensitive to agricultural management measures and environmental changes; thus, they can be used as indicators of soil quality and health^11,12^. Previous experiments showed that the application of straw and other organic fertilizers could increase the number of beneficial soil nematodes and decrease the number of phytophagous soil nematodes^13,14^. However, little information is available about whether and how the microenvironment soil conditions under straw mulching affect the structure of soil nematode communities, biodiversity and function in walnut orchards.

The major objectives of this study were to explore whether and how straw mulching affects the soil nematode community. Because straw is rich in nutrients and active substances, nutrients such as C and N can be released into the soil by a degradation pathway after straw mulching a walnut orchard, which provides a rich source of food for soil nematodes, thereby increasing soil nematode numbers and improving soil nematode community structure^15^. Therefore, we hypothesized that different straw mulching treatments could increase the number of nematodes and improve the community structure of soil nematodes. We also hypothesized that mixed-straw mulching would increase nutrient availability and improve soil fertility compared with rice straw mulching and rapeseed straw mulching because it would have a more suitable C/N ratio and faster degradation rate.

## Results

### Soil environmental conditions

*Mix straw* mulching treatments significantly correlated dissolved organic carbon (DOC) (*p* < 0.05) and NO_3_^–^N content (*p* < 0.05) (Table 1). In general, *mix straw* mulching and a cover distance of* n* (*Mix-n*) had a higher content of DOC and NO_3_^–^N than the other treatments (i.e., single-straw mulching). Meanwhile, the soil DOC content of the *Mix-n* treatment was significantly higher than that of the CK treatment. Though the content of NO_3_^–^N was higher in the *Mix-n* treatment than in the CK treatment, this difference was not significant. However, the soil pH, SMC and NH_4_^+^–N responses to different straw mulching treatments were not significant (*p* > 0.05).

**Table 1:** Overview of main effect of straw mulching quality and distance on environmental factors based on ANOVA.

Treatment	DOC mg kg^−1^	DON mg kg^−1^	NH_4_^+^–N mg kg^−1^	NO_3_^–^N mg kg^−1^	pH	SM (%)
Rice-n	73.84 ± 0.72ab	11.15 ± 0.52a	2.67 ± 0.23b	51.25 ± 5.46de	6.62 ± 0.27ab	24 ± 1.15a
Rice-1.5n	70.31 ± 1.04b	9.40 ± 0.60a	3.63 ± 1.11ab	38.35 ± 1.79e	5.97 ± 0.15bc	21.33 ± 0.33b
Rice-all n	75.12 ± 1.36ab	10.79 ± 1.02a	4.2 ± 0.30ab	66.69 ± 11.15abcd	6.47 ± 0.35abc	21.33 ± 0.33b
Rape-n	74.44 ± 0.59ab	10.44 ± 0.83a	4.58 ± 0.57ab	90.31 ± 9.77ab	6.49 ± 0.05abc	22.66 ± 1.2ab
Rape-1.5n	70.31 ± 0.39b	10.00 ± 0.60a	4.78 ± 0.50a	86.34 ± 11.59abc	6.3 ± 0.12abc	21.33 ± 0.33b
Rape-all n	71.48 ± 1.72b	10.24 ± 1.02a	4.16 ± 0.54ab	61.22 ± 1.52cde	6.58 ± 0.14abc	21.33 ± 0.33b
Mix-n	79.94 ± 4.57a	11.39 ± 0.97a	3.1 ± 0.25ab	93.56 ± 6.14a	6.54 ± 0.23abc	22.33 ± 0.66ab
Mix-1.5n	70.79 ± 1.67b	9.68 ± 0.09a	3.26 ± 0.60ab	64.97 ± 9.67bcd	6.79 ± 0.15a	21.66 ± 0.88ab
Mix-all n	75.79 ± 2.07ab	11.69 ± 0.41a	3.61 ± 0.68ab	73.84 ± 9.91abcd	6.46 ± 0.12abc	22 ± 0.57ab
CK	71.12 ± 0.75b	9.93 ± 1.04a	3.74 ± 0.20ab	81.68 ± 8.35abc	5.83 ± 0.38c	20.33 ± 0.88b
*p* value	< 0.05	> 0.05	> 0.05	< 0.05	> 0.05	> 0.05
F	2.73	0.97	1.34	4.58	1.71	1.78

Data are the means of three replicates ± SD .Within each column, the values with the same lower case letter are not significantly different. Different letters indicate statistically significant differences between treatments, according to the Duncan's multiple range test (*p* < 0.05). CK is no straw mulching treatment. The combination of *Rice-n, Rice-1.5n* and *Rice-all n* represents straw mulching types is *rice straw* and straw mulching distances is mean radius of crown width (*n*), one and half mean radius of crown width(*1.5 n*), and the whole quadrat(*all n*); The combination of *Rape-n, Rape-1.5n* and *Rape-all n* represents straw mulching types is rapeseed seed straw and straw mulching distances is *n*, *1.5 n* and *all n*; The combination of *Mix-n, Mix-1.5n* and *Mix-all n* represents straw mulching types is mixed rice and rapeseed seed straws and straw mulching distances is *n*, *1.5 n* and *all n*; similarly hereinafter.

### Soil nematode communities

The number of nematode genera in *Rice-n, Rice-1.5n, Rice-all n, Rape-n, Rape-1.5n, Rape-all n, Mix-n, Mix-1.5n, Mix-all n* and CK were 34, 37, 34, 31, 30, 26, 31, 30, 31 and 29, respectively (Appendix 1). Compared with those in other straw mulching treatments (*rice straw, rapeseed straw, and mix straw*), the total nematode genera were found to be significantly (*p* < 0.05) more numerous in the control treatment (CK) (Table 2). The control treatment (CK) and *mix-all n* treatment had significantly (*p* < 0.05) higher abundances of fungivores than the *Rapeseed -n* and *Rapeseed-1.5 n* treatments. Two *Rice straw* treatments (those with cover distances of n and all n) and one *Rapeseed straw* treatment (cover distance of 1.5n) had significantly (*p* < 0.05) lower abundances of plant parasites than the CK treatment. However, there were no significant differences among the different treatments in the abundance of omnivore-predator nematodes (Table 2).

**Table 2:** The abundances of total soil nematodes and trophic groups (means ± standard errors, n = 3) as affected by straw mulching treatments.

Treatment	Total nematode number/100 g dry soil	Bacterivore number/100 g dry soil	Fungivore number/100 g dry soil	Plant parasites number/100 g dry soil	Predator and omnivore number/100 g dry soil
Rice-n	178.1 ± 24.8de	69.1 ± 1.9b	48.8 ± 11.6abc	19.0 ± 0.9b	41.0 ± 12.2a
Rice-1.5n	292.2 ± 18.8bc	99.5 ± 9.3b	89.79 ± 43.5abc	61.0 ± 25.9ab	41.8 ± 17.0a
Rice-all n	199.9 ± 7.4de	54.2 ± 17.7b	73.9 ± 3.1abc	16.3 ± 3.9b	55.4 ± 10.7a
Rape-n	168.8 ± 11.2e	71.4 ± 13.4b	18.6 ± 12.3b	43.1 ± 6.6ab	35.5 ± 9.0a
Rape-1.5n	167.7 ± 17.6e	83.2 ± 10.0b	26.4 ± 11.2b	19.2 ± 7.1b	38.8 ± 6.6a
Rape-all n	286.9 ± 41.6bc	88.2 ± 32.3b	105.1 ± 22.4ab	40.4 ± 18.5ab	53.0 ± 15.9a
Mix-n	256.6 ± 32.2bcd	96.1 ± 24.9b	55.9 ± 28.6abc	52.7 ± 9.8ab	51.7 ± 9.4a
Mix-1.5n	210.2 ± 38.3cde	60.4 ± 7.1b	51.1 ± 32.7abc	41.5 ± 8.7ab	57.0 ± 7.0a
Mix-all n	300.3 ± 31.4b	81.7 ± 28.3b	115.4 ± 9.8a	40.2 ± 5.3ab	62.9 ± 17.3a
CK	410.7 ± 8.1a	174.1 ± 13.3a	119.1 ± 42.1a	74.3 ± 35.5a	43.2 ± 4.4a

Different letters indicate statistically significant differences between treatments, according to the Duncan's multiple range test (*p* < 0.05).

The *Rapeseed-1.5n* and *Mix-1.5n* treatments had significantly higher omnivore-predator footprints and structure footprints than the *Rice-n* and *Mix-all n* treatments (*p* < 0.05) (Table 3). The metabolic footprints of fungivores were higher under *Rapeseed-all n* and *Mix-all n* than under the other treatments, while the metabolic footprint of bacterivores had greater values in the *Rapeseed-1.5n* treatment than in other treatments. There was no obvious change in the plant parasite footprint or the enrichment footprint among different treatments.

**Table 3:** Soil nematode metabolic footprints (μg C kg^−1^ soil) (means ± SE).

Treatment	efootprint	sfootprint	PP footprint	FF footprint	BF footprint	OP footprint
Rice-n	4.93 ± 1.83a	10.82 ± 3.05c	3.63 ± 1.40a	2.21 ± 0.13ab	7.38 ± 2.11ab	12.95 ± 4.03c
Rice-1.5n	5.72 ± 1.56a	18.29 ± 4.17abc	4.93 ± 1.40a	2.62 ± 1.04ab	7.55 ± 1.72ab	23.89 ± 7.58bc
Rice-all n	4.43 ± 0.94a	21.89 ± 7.71abc	5.02 ± 3.18a	3.12 ± 0.34ab	5.15 ± 0.89b	33.7 ± 15.51abc
Rape-n	5.29 ± 3.07a	18.20 ± 1.30abc	6.36 ± 1.21a	1.07 ± 0.42b	9.29 ± 3.22ab	28.15 ± 0.35abc
Rape-1.5n	8.7 ± 5.21a	30.48 ± 6.44ab	5.83 ± 4.17a	1.68 ± 0.55ab	13.74 ± 5.44a	48.92 ± 11.55ab
Rape-all n	4.00 ± 0.44a	21.15 ± 1.34abc	5.36 ± 3.73a	3.53 ± 0.66a	4.11 ± 0.88b	34.09 ± 4.34abc
Mix-n	2.85 ± 1.30a	20.09 ± 5.92abc	6.28 ± 2.09a	1.83 ± 0.50ab	7.40 ± 1.88ab	26.63 ± 7.94abc
Mix-1.5n	2.80 ± 1.73a	34.14 ± 6.07a	7.82 ± 4.26a	2.14 ± 1.08ab	5.49 ± 0.67b	55.42 ± 11.30a
Mix-all n	5.54 ± 1.37a	10.57 ± 3.10c	7.05 ± 3.15a	3.43 ± 0.49a	6.10 ± 1.19ab	11.11 ± 3.47c
CK	7.17 ± 2.70	16.67 ± 4.31bc	3.52 ± 1.73a	2.51 ± 0.75ab	9.09 ± 1.28ab	26.80 ± 8.82abc
*p* value	0.79	0.048	0.986	0.245	0.279	0.037
F	0.59	2.421	0.231	1.419	1.339	2.59

Different letters indicate statistically significant differences between treatments, according to the Duncan's multiple range test (*p* < 0.05).

### Soil nematode faunal profile

**Figure 1:**
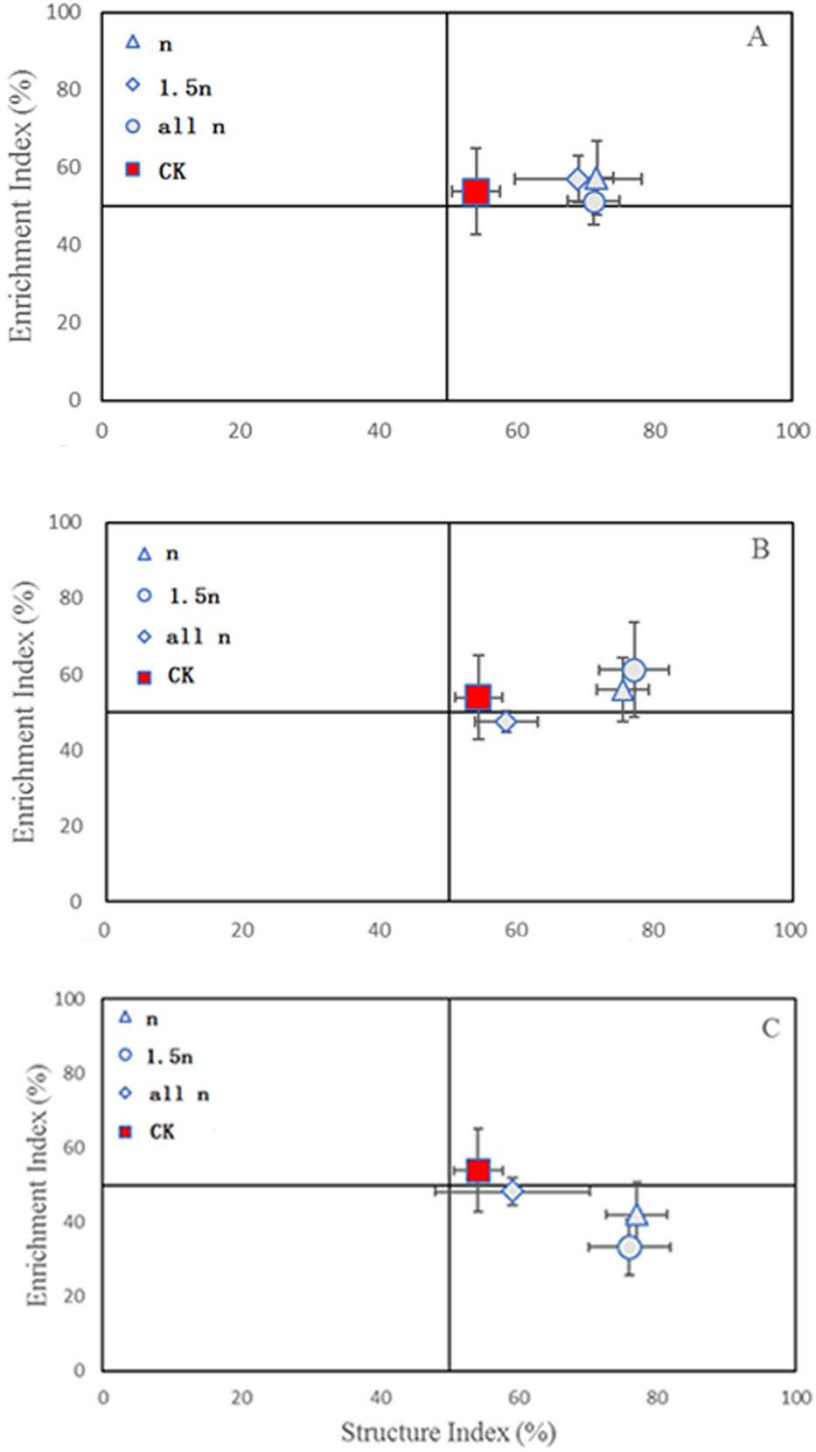
Distribution map of soil nematode flora under different straw mulching treatments (**A** representing rice straw pattern; **B** representing rape straw pattern; **C** representing mixed straw pattern).

The soil food webs of the straw mulching treatments were plotted along their respective SI and EI trajectories in Fig. 1. A discernible pattern was found in the nematode faunal profile of different straw mulching treatments. The nematode fauna analysis showed that the all different coverage distance of the *rice straw* treatment and the coverage distances were *n* and *1.5n* of the *rapeseed straw* treatment was distributed in the B quadrant (Fig. 1A,B). While, when the coverage distance was increased to cover the whole plot (*all n*) of the *rapeseed straw* treatment, it was distributed in the C quadrant. For the *mix straw* treatment, all coverage distances were distributed in the C quadrant. The variation in SI value increasing coverage distance is not obvious. EI value tends to decrease with increasing coverage distance, and the distribution of the straw mulching treatments gradually approaches the C quadrant level from the B quadrants (Fig. 1).

### Nematode diversity

**Figure 2:**
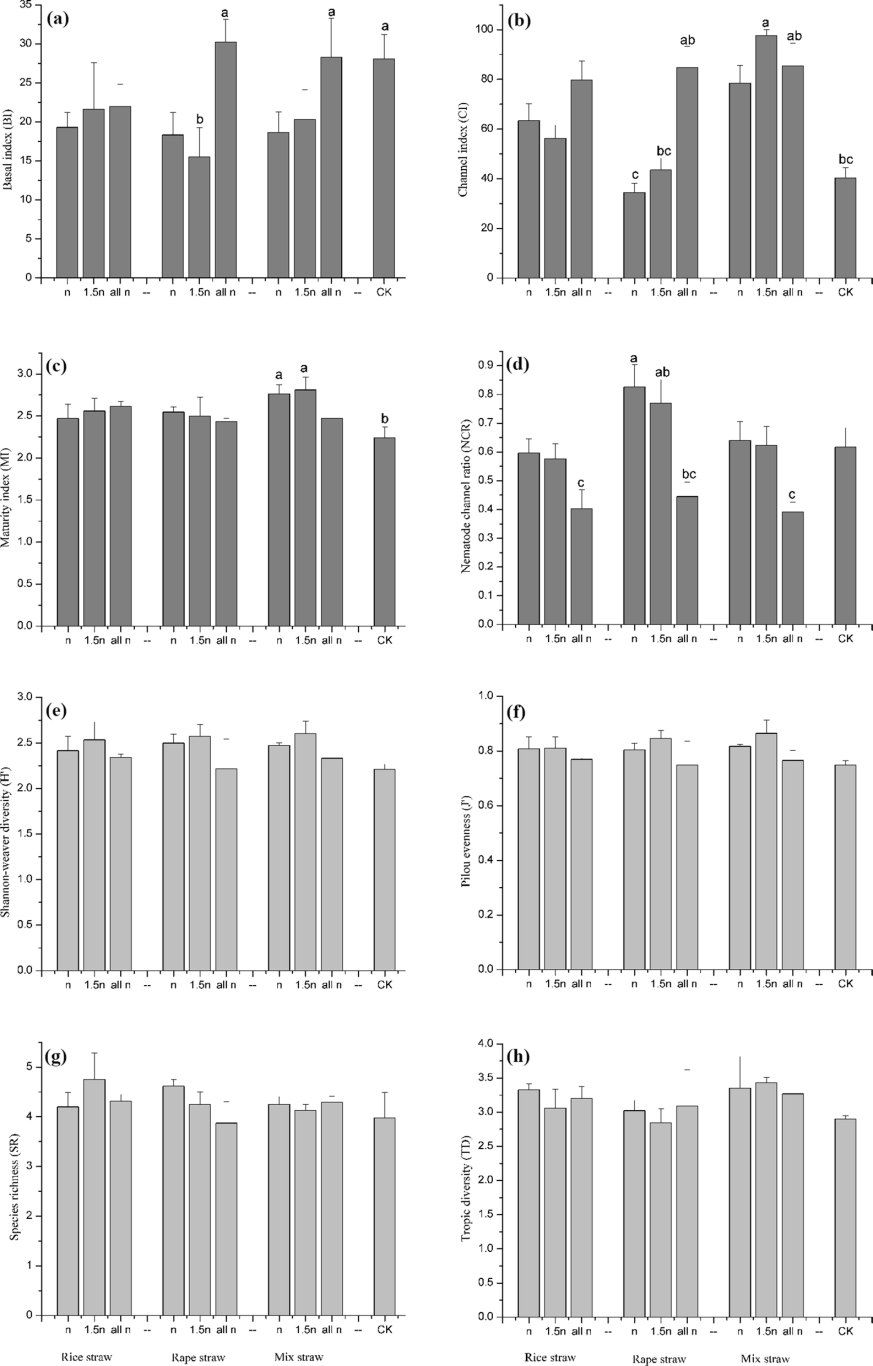
Changes of soil nematode ecological index under different straw. (**a**) Effect of straw mulching on Basal index, (**b**) channel index, (**c**) maturity index, (**d**) nematode channel ratio, (**e**) Shannon–Weaver index, (**f**) Pielou’s evenness, (**g**) species richness index, (**h**) trophic diversity index; means ± S. (*p* < 0.05).

Significant differences were observed in the basal index (BI), channel index (CI), and maturity index (MI) between the different straw mulching treatments. MI and CI were significantly (*p* < 0.05) higher in the *mix straw* mulching treatment than in the CK treatment, especially in the mixed treatment with straw mulching distance at *1.5n* (Fig. 2a–c). For all straw treatments, BI and CI were higher for the whole plot mulching *(all n*) than for the other mulching distances (*n, 1.5n*). Moreover, there were no fluctuations among different treatments in terms of the Shannon–Weaver index (H^’^), Species richness index (SR), Trophic diversity index (TD) or Pielou’s evenness index (J^’^) (Fig. 2d–g).

### Environmental factors affecting soil nematode community variability

Under different straw mulching treatments, DON had a significant (*p* < 0.05) negative correlation with plant parasite nematodes (Table 4). NH^4+^–N was significantly (*p* < 0.05) negatively correlated with omnivore-predator nematodes. SM had a significant (*p* < 0.05) negative correlation with the total nematode abundance. However, DOC, NO_3_^–^N, and pH had no significant correlations with the soil nematode communities (Table 4).

**Table 4:** Relationships between nematode abundances and environmental factors based on Pearson correlation.

Factor	Total	BF	FF	PP	OP
DOC	.058	.087	− .073	− .010	.239
DON	− .087	− .037	.083	− .417*	.124
NH_4_^+^–N	.005	.104	.094	− .057	− .365*
NO_3_^–^N	− .017	.074	− .126	.018	.071
pH	− .275	− .279	− .096	− .334	.180
SM	− .412*	− .237	− .337	− .238	− .024

*p < 0.05.

## Discussion

### Soil environmental conditions

It is clear that the *Mix-n* treatment had higher DOC and NO_3_^–^N than the other treatments under all soil environmental conditions. Due to the different C/N ratios of the different straw types, N degradation and mineralization were also different. The change in soil nutrients caused by straw mulching is mainly due to the role of soil organisms. Therefore, we can explain the difference in soil nutrients by the soil biological composition of different straw mulching treatments. In general, the specific genus of soil nematode in the *mix* treatment can characterize the particular soil nutrient status. Previous studies have shown that some nematodes are found more often in areas with similar environmental variables and that nematode genera within the same trophic group responded differently to environmental variables^16^. We found that the higher abundances of *Prismatolaimus*, *Cephalobus* and *Eucephalobus* corresponded to the higher soil NO_3_^–^N (Appendix 1). Our results are consistent with the observations of Song et al.^17^. Moreover, the *Mix-n* treatment had a higher density of *Mesodorylaimus*, *Aphelenchoides* and *Thonus* where the DOC was higher. This result is in agreement with the findings of Olatunji et al.^18^, in which *Thonus*, *Aporcelaimus*, *Mesodorylaimus*, *Aphelenchoides*, *Criconemoides*, *Tylenchus*, and *Rhabditidae* were positively associated with DOC.

### Soil nematode communities

From the data in Table 2, it is apparent that the CK treatment had a higher total number of soil nematodes and a higher abundance of soil nematodes in different nutritional groups than any straw mulch treatment; that is, the number of soil nematodes after straw mulching was lower than that in the control. Blankinship et al.^19^ used a meta-analysis method to study the response of soil nematodes to temperature increase under different ecosystem types. It was found that soil nematodes were mainly affected by annual precipitation. When annual precipitation exceeded 626 mm, the increase in temperature had a positive effect on the number of soil nematodes^19^. In this study, the annual precipitation in this area (1033.9 mm) exceeded 626 mm, and straw mulching had a cooling effect during the growth period of young walnut trees. This could be a possible reason of higher abundance of soil nematodes in the CK treatment than that in any straw mulching treatment. Moreover, this finding is also contrary to our first hypothesis that different straw mulching treatments would increase the number of soil nematodes. The reasons are as follows: on the one hand, phenolic acids enter the soil through the secretions of walnut roots and the decomposition of a large amount of straw residues, which results in an increase in phenolic acids in the soil and a decrease in the total number of soil nematodes and other nematodes^20^. On the other hand, straw mulching returns pathogenic bacteria and parasite eggs to the field directly. At the same time, the nutrients released from straw in the soil provide a favorable environment for pathogenic bacteria and parasite eggs to increase in number, which significantly inhibits soil nematodes^21^.

In addition, a key finding was that fungal nematodes were more common than bacterial nematodes in the treatments with complete mulch coverage than in the *n* and *1.5n* coverage treatments. When *rice straw, rapeseed straw* and *mix straw* were applied at *n* and *1.5n* distances, the decomposition pathway was a bacterial channel; when the coverage distance increased to *all n*, the decomposition pathway gradually changed to decomposition equally distributed between bacterial and fungal decomposition pathways. In contrast, the CK treatment was dominated by the number of bacterivorous nematodes, suggesting that the bacterial channel was the main pathway of decomposition, which was consistent with the result of the distribution map of nematode fauna in Fig. 1. At the same time, this result indicates that the coverage distance changed the dominant community of nematode trophic groups.

The footprints of different nematode trophic groups are proxies for the carbon or energy flow entering the soil food web through their respective channels^22^. In our study, we found that the footprint and the carbon biomass of the omnivore-predator nematodes and all structure metabolic footprints showed higher values under all straw mulching treatments compared with those of the other soil nematode trophic groups (Table 3). This observation may be explained by the predator–prey trophic cascade effect: straw mulching stimulates higher carbon and nutrient inputs first to microorganisms and then to microbivorous nematodes, which stimulate the metabolic activity and abundance of omnivore-predator nematodes; omnivore-predator nematodes consume more prey and thus inhibit the abundance of soil nematodes at lower trophic levels^23^.

### Nematode diversity

The maturity index of nematodes is one of the key indices of soil health. In our study, the MI values for *rice straw* and *rapeseed straw* treatment alone were not significantly higher than those for the CK treatment (Fig. 2c). However, the MI values for the *mix straw* treatments were significantly higher than those for the CK treatment, indicating that the structure of the nematode community is stable and that the complexity of the soil food web could increase under the *mix straw* treatment.

Combined with the ecological indices BI, which is related to soil properties and decomposition pathways^24^, we found that higher CI value for the three straw mulching treatments appeared in the whole-plot coverage treatments (*all n*). Our results contrast with those of other studies, which found that bacterial-dominated decomposition pathways were the most common pathways^20^. This discrepancy could be explained mainly by the observed variations in the abundances of bacterivores and fungivores among the different coverage distances. Specifically, bacterivore nematodes predominate in different soil nematode trophic groups when the coverage distance is *n*, while bacterivore nematodes and fungivore nematodes predominate in different soil nematode trophic groups when the coverage distance is increased to *all n* (Table 2). In addition, soil nematode decomposition pathway changed with the increase in coverage distance in the three straw mulching treatments, which may have been caused by the increase in contact area between straw and soil. The specific mechanism needs to be further studied in our next work.

### Soil nematode faunal profile

The SI is considered to indicate the structure of the soil food web response to disturbance and during remediation, while the EI reflects soil food web responses to available resources and the resource response to the primary decomposers^17,25^.

In the present study, the *rice straw* mulching treatments and *rapeseed straw* mulching treatments with high EI and SI values at different straw mulching distances were in quadrant B, indicating that the structure of the food web was fairly mature, the N concentration was high, the C:N ratio was low, the decomposition pathways of fungi and bacteria was balanced, and the disturbance level of the soil environment was low to moderate. These conditions occurred is mainly because of the large amounts of dissolved organic carbon and dissolved organic nitrogen in the soil due to straw degradation and the straw mulching water retention effect making the soil moisture content higher than that found in the CK treatment (Table 1).

However, the *mix straw* mulching treatments with high SI and low EI values at different straw mulching distances were in quadrant C, which indicates a structured food web, medium soil enrichment, a moderately high C/N ratio, fungal decomposition channels, and no disturbance. Our previous research suggested that the *mix straw* mulching treatment had a moderate carbon nitrogen ratio (C:N) and that *mix straw* degrades more quickly than *rice straw* or *rapeseed straw*^9^. In addition, the *mix straw* may have provided stable moisture content and higher dissolved organic carbon and dissolved organic nitrogen than *rice straw* or *rapeseed straw* (Table 1), thus increasing nutrient availability and soil fertility levels. This result is supported by other agricultural management practices^20,26,27^. This evidence supported our hypothesis that the *mix straw* mulching treatment led to a more stable soil food web and higher soil fertility levels.

### Environmental factors affecting soil nematode community variability

Straw mulching directly increases the mineral nitrogen and DON contents in the soil through decomposition, which significantly increases the content of nitrogen in the soil, thus increasing the amount of soil nutrients and soil organisms. Plant parasite and omnivore-predator nematode abundances were negatively correlated with NH^4+^–N and DON contents, but there was no significant correlation between the nematode community and soil DOC content. This finding indicates that nitrogen in the soil of the agroforestry ecosystem had a more significant impact on the nematode community than carbon. This result is also consistent with previous results^28,29^. Another possible explanation was that ammonium toxicity may occur when soil nematodes feed on root fluid, resulting in a negative correlation between omnivore-predator nematodes and NH^4+^–N^30^. Compared with the control condition, straw mulching significantly increased soil moisture content and soil anoxia, while soil total nematodes were negatively correlated with SM value. The results showed that the increase in soil moisture changed the soil environment, inhibited the growth of soil microorganisms, and inhibited the growth of total nematodes through changes in nutrient levels and the environment in the food chain.

In terms of straw coverage distance, our results showed that the decomposition pathway gradually changed from the bacterial decomposition channel to the bacterial/fungal decomposition channel when the coverage distance increased from a narrow coverage distance (*n*) to a wide coverage distance (*all n*) in the three straw mulching treatment groups. In terms of straw mulch types, the *mix straw* mulching treatment had a higher maturity index, a more stable soil food web and higher soil fertility levels than the *rice straw* or *rapeseed straw* mulching treatments. There was a significant negative correlation between plant parasite and omnivore-predator nematodes and NH^4+^–N and DON, but there was no significant correlation between the nematode community and the soil DOC content. This finding was unexpected and suggests that nitrogen in the soil of agroforestry ecosystems had a more significant impact than soil carbon on the nematode community. Recommendations for sustainable walnut orchard management based on the complexity and stability of nematode food webs should advocate the use of *mix straw* mulching (*mix*) covering the whole plot (*all n*) and thus promote the accumulation of soil dissolved organic nitrogen and carbon nutrients.

## Materials and methods

### Experimental site

The study was conducted in a large walnut orchard field in Langzhong (31° 57′ 82″ N, 105° 96′ 65″ E; 712.5 m above sea level), which is the hilly area of the central Sichuan Basin, southwestern China. The area has a humid mid-subtropical monsoon climate, with an average annual precipitation of 1033.9 mm and an annual temperature of 18.7 °C. This site has purple soil, classified as Pup-Orthic Entisol in the Chinese Soil Taxonomy (CST) and Entisol in the USDA Soil Taxonomy^31^. The specific soil in this study was a loam soil with the following nutrient profile (0–15 cm depth): total nitrogen (2.4 g kg^−1^), available phosphorus (0.96 g kg^−1^), available potassium (86.57 mg kg^−1^) and total carbon (5.95 g kg^−1^)^32^.

### Experimental design

The walnut sapling (*Juglans regia*) plantation covered a 30 m × 90 m area, with a southerly slope of c. 2.5 degrees. The walnut saplings were planted in April 2010 and then grafted in May 2015.

In July 2016, we established a straw mulching experiment with a randomized block design in a walnut plantation with 3 m × 3 m spacing to investigate the potential effect of straw mulching on nematode community abundance and diversity and the associated agroecosystem function. We selected three different straw mulch types, *rice straw*, *rapeseed straw,* and *mix straw* (of equal quality, mixed 1:1), as the main plot. Then, under different main plots, we set up three different straw mulching distances (covering the mean radius of the crown width (*n*), covering 1.5 times the mean radius of the crown width (*1.5 n*) and covering the whole experimental plot (*all n*)) as sub-plots. Plots with no straw mulching were used as the CK plots. The quantity of straw mulch in each treatment was 3 kg/m^2^, selected based on previous research results^33,34^. There were a total of 10 treatments, based on the three straw mulching types, the three different straw mulching distances and CK, and each treatment had three replicates. All treatments were subjected to random permutations.

### Soil sampling and property analysis

Soil samples were collected on 19 October 2016. Five soil samples from the 0–20 cm soil layer were taken with a soil auger (Φ = 2.5 cm) by using the five-spot method. The samples were combined to form one composite sample per plot location. Each composite sample was sieved (2 mm) and stored in individual plastic bags, immediately transferred to a cold room with a temperature of 4 °C, and then processed within a week. The samples used to analyze pH, SMC, NH_4_^+^–N, NO_3_–N, DON and DOC were air-dried at room temperature.

### Analysis of soil physicochemical properties

Soil moisture content was estimated gravimetrically by oven drying 20 g of each field composite soil sample at 105 °C for 24 h. Soil pH was determined with deionized water and an air-dried and fine-ground sample at a ratio of 1:2.5 (weight to volume, w/v) with an electronic pH meter. Soils were extracted with 2 M KCl, and the filtrate was analyzed for NH_4_^+^–N and NO_3_^–^N content (with an Acquity Ultra-Performance Liquid Chromatograph, AA3, Bran + Luebbe, Germany). Dissolved organic carbon (DOC) and nitrogen (DON) were estimated using a TOC/TN analyzer (Multi N/C 2100(S), Analytik Jena AG, Germany).

### Nematode extraction and identification

Soil nematodes were extracted from 50 g of fresh soil using a modified cotton-wool filter method^35^. The extractions were used for identification (at least 100 nematodes) at the genus level using a microscope (OLYMPUS BX51) at 100 × magnification (resolution: 0.25 μm) according to Ahmad et al.^36^. If fewer than 100 nematodes were observed in one sample, all specimens were identified. Nematode abundance was adjusted according to soil moisture and was expressed as the number of nematodes per 100 g dry soil. After identification (within one week), based on their feeding habits, nematodes were classified into four trophic groups: (1) bacterivores (Ba), (2) fungivores (Fu), (3) omnivores-predators (OP) and (4) plant parasites (PP)^12^.

The assumed effects of straw mulching on soil nematodes were examined with the following variables: (1) total nematode abundance; (2) abundance of individual trophic groups including PP, Ba,Fu,OP;(3)Shannon–Weaver index (H^’^)^37^; (4) Pielou’s evenness index (J^’^)^38^; (5) maturity index (MI); (6) trophic diversity index (TD)^39^; (7) species richness index (SR)^11^, and (8) basal index (BI)^40^.

The metabolic footprint approach uses existing data on nematode biovolumes and growth rates, and the weightings used in the enrichment index (EI), structure index (SI), and channel index (CI) calculations^41^ to estimate the C metabolism of the nematode community^22^. The nematode metabolic footprints (NMF) was also divided into the enrichment footprint (efootprint), representing lower trophic levels (c-p 1–2), and the structure footprint (sfootprint), representing higher trophic levels (c-p 3–5). The above data were calculated using the online Nematode Indicator Joint Analysis (NINJA) tool^42^.

### Data analysis

The nematode abundances were ln (x + 1) transformed prior to statistical analysis for the normality of data. One-way ANOVA was used to test the effect of straw mulching on soil properties, nematode abundance, and nematode ecological index under each treatment. Correlation analyses between abiotic and biotic drivers, including pH, SMC, NH_4_^+^–N, NO_3_^–^N, DON, DOC and nematode community data, were conducted. Statistical significance was determined at *p* < 0.05. Differences between data means were analyzed with t-tests using SPSS v. 17.0 (SPSS Inc., Chicago, IL) statistical software. Least significant difference (LSD) was used to test for differences among treatment means.

## Supplementary information

Supplementary file1
